# Multiplex Detection of *Aspergillus fumigatus* Mycoviruses

**DOI:** 10.3390/v10050247

**Published:** 2018-05-08

**Authors:** Selin Özkan-Kotiloğlu, Robert H. A. Coutts

**Affiliations:** 1 Department of Molecular Biology and Genetics, Ahi Evran University, Kırşehir 40100, Turkey; 2 Department of Life Sciences, Faculty of Natural Sciences, Imperial College London, London SW7 2AZ, UK; r.coutts@herts.ac.uk; 3 Department of Biological and Environmental Sciences, University of Hertfordshire, Hatfield AL10 9AB, UK

**Keywords:** dsRNA mycoviruses, multiplex PCR, Aspergillus fumigatus chrysovirus, Aspergillus fumigatus partitivirus-1, Aspergillus fumigatus tetramycovirus-1.

## Abstract

Mycoviruses are viruses that naturally infect and replicate in fungi. They are widespread in all major fungal groups including plant and animal pathogenic fungi. Several dsRNA mycoviruses have been reported in *Aspergillus fumigatus*. Multiplex polymerase chain reaction (PCR) amplification is a version of PCR that enables amplification of different targets simultaneously. This technique has been widely used for detection and differentiation of viruses especially plant viruses such as those which infect tobacco, potato and garlic. For rapid detection, multiplex RT-PCR was developed to screen new isolates for the presence of *A. fumigatus* mycoviruses. Aspergillus fumigatus chrysovirus (AfuCV), Aspergillus fumigatus partitivirus (AfuPV-1), and Aspergillus fumigatus tetramycovirus-1 (AfuTmV-1) dsRNAs were amplified in separate reactions using a mixture of multiplex primer pairs. It was demonstrated that in the presence of a single infection, primer pair mixtures only amplify the corresponding single virus infection. Mixed infections using dual or triple combinations of dsRNA viruses were also amplified simultaneously using multiplex RT-PCR. Up until now, methods for the rapid detection of Aspergillus mycoviruses have been restricted to small scale dsRNA extraction approaches which are laborious and for large numbers of samples not as sensitive as RT-PCR. The multiplex RT-PCR assay developed here will be useful for studies on determining the incidence of *A. fumigatus* mycoviruses. This is the first report on multiplex detection of *A. fumigatus* mycoviruses.

## 1. Introduction

*Aspergillus* is a genus in the phylum Ascomycota, kingdom Fungi. *Aspergillus* species (spp.) are universal and ubiquitous in nature, and are usually found as saprophytes in soil, decaying vegetation, seed and grains [1]. They are important both medically and commercially. *Aspergillus fumigatus* is an important pathogen with the capability of infecting humans, invading lungs and leading to opportunistic infections in immunocompromised hosts along with the allergic reactions in healthy individuals [1,2].

Mycoviruses were first reported in *Aspergillus foetidus* and *Aspergillus niger* [3]. Research on dsRNA elements showed that various *Aspergillus* spp. were infected by mycoviruses [4] (Varga et al., 1998). Previously, research on 61 isolates of *A. fumigatus* showed that none of the strains contained dsRNA elements [4]. However, after screening more than 360 *A. fumigatus* isolates from environmental and clinical sources for the presence of dsRNA elements, three different dsRNA profiles were reported with a 6.6% incidence in the UK isolates [5]. Recently, different dsRNA mycoviruses have been reported in *A. fumigatus* with an 18.6% incidence in Holland [6]. Those previous studies reporting variable mycovirus incidence in *A. fumigatus* have employed common nucleic acid extraction methods [4,5,6]. The impacts of the viruses on the host were also investigated in *A. fumigatus* using murine and *Galleria mellonella* models and those viruses are known to affect host virulence [7,8]. 

Multiplex detection technologies where simultaneous detection of multiple viruses in a single assay can be achieved, enables a reduction in cost, time and labor as compared to other detection methods [9]. Various methods such as enzyme-linked immunoabsorbant assay (ELISA), real-time polymerase chain reaction (RT-PCR), loop-mediated isothermal amplification (LAMP), Luminex bead arrays and next generation sequencing (NGS) have all been used for virus detection [9,10,11,12]. Multiplex PCR is a version of PCR, which facilitates amplification of several different target sequences simultaneously. In this technique, more than one pair of primers is used to amplify different targets in one reaction. Multiplex PCR is a useful tool to detect various viruses infecting the same sample [10,13]. It is called multiplex reverse transcription PCR (RT-PCR) when the starting material is RNA instead of DNA. This technique has been widely used for the detection and differentiation of the viruses especially plant viruses such as tobacco, potato and garlic viruses [10,13,14,15,16]. 

The main aim of this study was to optimize a rapid detection method for three characterized *A. fumigatus* mycoviruses namely Aspergillus fumigatus chrysovirus (AfuCV) [17], Aspergillus fumigatus partitivirus (AfuPV-1) [18], and Aspergillus fumigatus tetramycovirus-1 (AfuTmV-1) [19], which have been reviewed recently by Kotta-Loizou and Coutts [20]. For rapid detection, multiplex PCR was developed to screen new isolates in terms of presence of *A. fumigatus* mycoviruses. 

## 2. Materials and Methods 

### 2.1. Fungal Growth Conditions and Mycoviruses 

*A. fumigatus* strains used in this study were isolates A56, A88 and Af293 for Aspergillus fumigatus chrysovirus (AfuCV), Aspergillus fumigatus partitivirus-1 (AfuPV-1) and Aspergillus fumigatus tetramycovirus-1 (AfuTmV-1), respectively [5]. All *A. fumigatus* strains were cultured from glycerol stocks on Aspergillus complete medium (ACM) agar at 37 °C for 3–4 days and spores were harvested in order to inoculate ACM broth which was incubated at 37 °C with shaking at 130 rpm. After 5 days, mycelia were harvested using sterile Miracloth (Merck Millipore, MA, USA), rapidly frozen in liquid N_2_ and kept at −80 °C until processing. *A. fumigatus* isolates were selected from the ones screened previously by Bhatti et al., 2012 [5].

### 2.2. Primer Design

Sequence-specific oligonucleotide primers were designed for amplification of conserved areas within the *RdRP* genes of AfuCV, AfuPV-1 and AfuTmV-1. The *RdRP* gene sequences of each mycovirus were obtained from the National Center for Biotechnology Information (NCBI). Specificity of the primers was checked by BLAST analysis of mycoviral genomes and the *A. fumigatus* genome. In order to facilitate correct multiplex PCR amplification, all primers were designed with similar annealing temperatures. The sequences of the oligonucleotide primers used are shown in Table 1. 

### 2.3. RNA Extraction and Reverse Transcription

In order to perform multiplex PCR for mycovirus-infected *A. fumigatus* isolates, total fungal RNA was extracted using the RNeasy Plant Mini kit (Qiagen, Hilden, Germany) from 100 mg of grounded mycelium, quantified by using NanoDrop 2000C spectrophotometer (Thermo Fischer, Waltham, MA, USA) and cDNA was synthesized using Superscript-III first-strand synthesis system (Invitrogen, Carlsbad, CA, USA) according to the manufacturers’ protocol as follows; 5 µg of RNA, 10 mM of dNTP mix and 250 ng of random primers along with 6.5 µL of DEPC-treated water were incubated at 65 °C for 5 min and snap cooled on ice for at least 2 min. Then 4 µL of 5× first strand buffer, 0.1 M DTT, 40 U of RNasin RNase inhibitor (Promega, Madison, WI, USA) and 200 U of Superscript-III reverse transcriptase were added to the reaction after a brief centrifugation. The 20 µL reaction mixture was then subjected to the following cycling regime of incubation at 25 °C, 50 °C and 70 °C for 5 min, 1 h and 15 min, respectively in a DNA Engine DYAD thermocycler. In an attempt to confirm if there was direct amplification from DNA or not, cDNA synthesis was performed without the reverse transcriptase enzyme. Additionally, viral dsRNAs were extracted using LiCl extraction as described previously [21] and used as a positive control in order to check the efficiency of the kit at extracting viral dsRNAs. 

### 2.4. Specificity and Sensitivity Testing of Single PCR and Multiplex PCR

Single PCR amplification was performed prior to multiplex PCR in order to check the primers and amplicon sizes. A no template control was included along with the specificity tests consisting of (i) a mixture of the three primer pairs and cDNA template from each virus, and (ii) a mixture of the three primer pairs and the mixture of three viruses (2 ng from each mycovirus). The PCR master mix contained 10 µL of 5× GoTaq Buffer (Promega, Fitchburg, WI, USA), 1 µL of 100 mM dNTP mix (Promega), 3 µL of forward primer mixture (10 µM of each primer), 3 µL of reverse primer mixture (10 µM of each primer), 2 U of GoTaq polymerase (Promega) and 5 ng DNA or cDNA template in 50 µL reaction. 

Multiplex PCR was performed using a program consisting of an initial activation at 95 °C for 3 min followed by 32 cycles of 3 steps including 95 °C for 30 s, 62 °C for 45 s and 72 °C for 30 s and a final extension at 72 °C for 3 min. The resulting PCR amplicons were analyzed by electrophoresis in 2% agarose gel containing ethidium bromide as before [20].

## 3. Results

### 3.1. The Efficiency of the RNA Extraction Method

**Figure 1:**
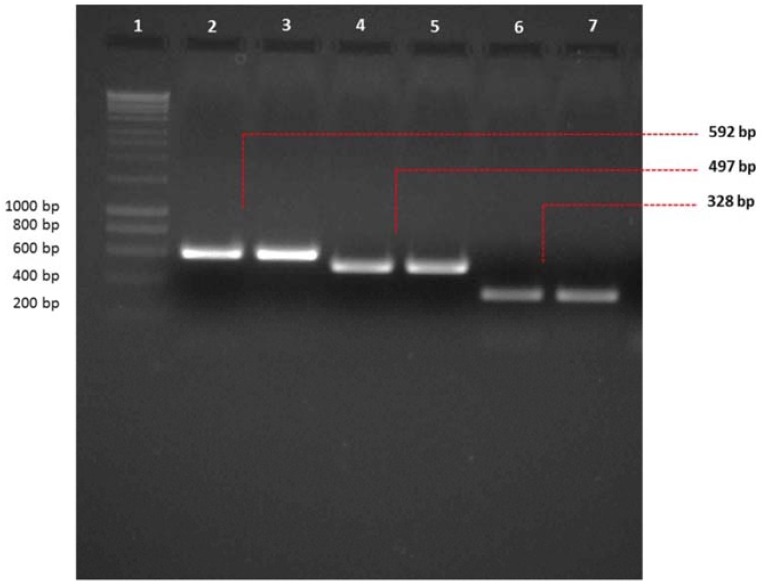
Conventional PCR to check amplicon size prior to multiplex PCR. Amplicon sizes were checked on two percent agarose gel prior to performing multiplex PCR for *A. fumigatus* dsRNA mycoviruses. AfuCV, AfuPV-1 and AfuTmV-1 dsRNAs were extracted using LiCl extraction (Lanes 2, 4 and 6, respectively) and RNeasy Plant Mini kit (Lanes 3, 5 and 7, respectively) and used as templates for amplification with AfuCV, AfuPV-1 and AfuTmV-1 primers (2–3, 4–5, 6–7, respectively). Hyperladder-I was used as a marker to estimate the size of the amplicons (Lane 1).

The multiplex RT-PCR amplification assay was used to detect known and characterized *A. fumigatus* mycoviruses, namely AfuCV, AfuPV-1 and AfuTmV-1. The effect of the dsRNA extraction method on the efficiency of PCR was tested using LiCl extraction and the RNeasy Plant Mini kit (Qiagen). It was found that both gave the same amplicon with similar efficiency (Figure 1). We demonstrated that targets of interest can be amplified with the same efficiency from viral dsRNAs obtained using different procedures such as virus purification or an RNA extraction kit. 

### 3.2. Specificity and Sensitivity of the Multiplex PCR

**Figure 2:**
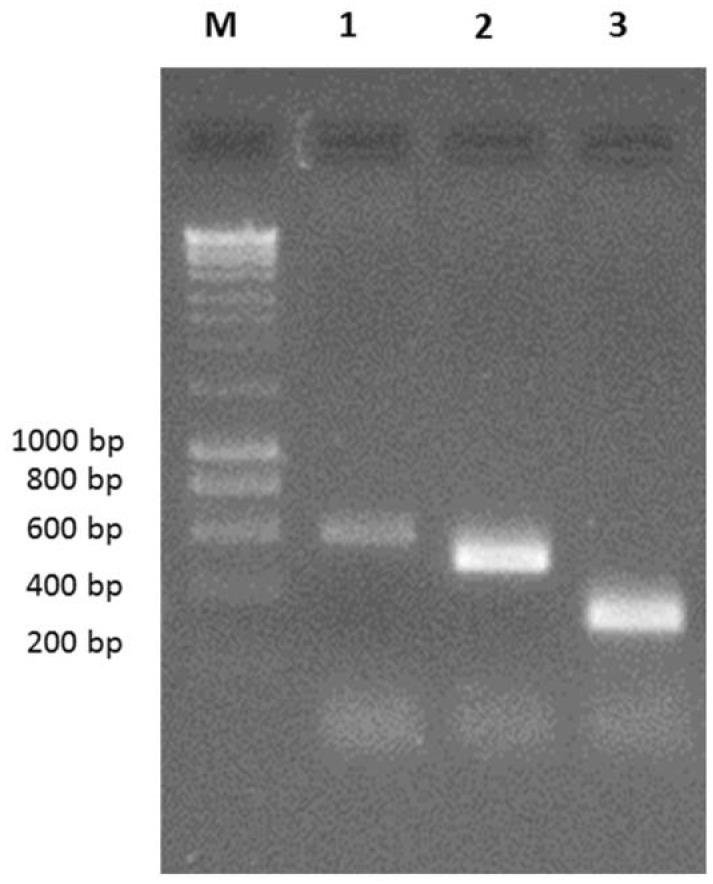
Specificity testing of multiplex RT-PCR with a mixture of three primer pairs. Multiplex RT-PCR was performed using the mixture of three primer pairs in a reaction including single template. Lanes 1, 2, 3 indicate the amplicons from AfuCV, AfuPV-1 and AfuTmV-1 templates in the presence of mixed primers, respectively. Hyperladder-I was used as a marker to estimate the size of the amplicons (Lane M).

After establishing the dsRNA extraction method, primers were examined in terms of specificity under multiplex conditions. AfuCV, AfuPV-1 and AfuTmV-1 dsRNAs were amplified in separate reactions using a mixture of multiplex primer pairs. It was determined that in the presence of single infection, mixtures of primer pairs only amplify the virus corresponding single infection (Figure 2). The specificity of the mixture of three primer pairs used in the present study was tested and shown in Figure 2. As positive controls oligonucleotide primer pairs were used for RT-PCR amplification of amplicons 592, 497 and 328 bp in size from respectively AfuCV, AfuPV-1 and AfuTmV-1. No cross-reaction with non-targets was identified.

**Figure 3:**
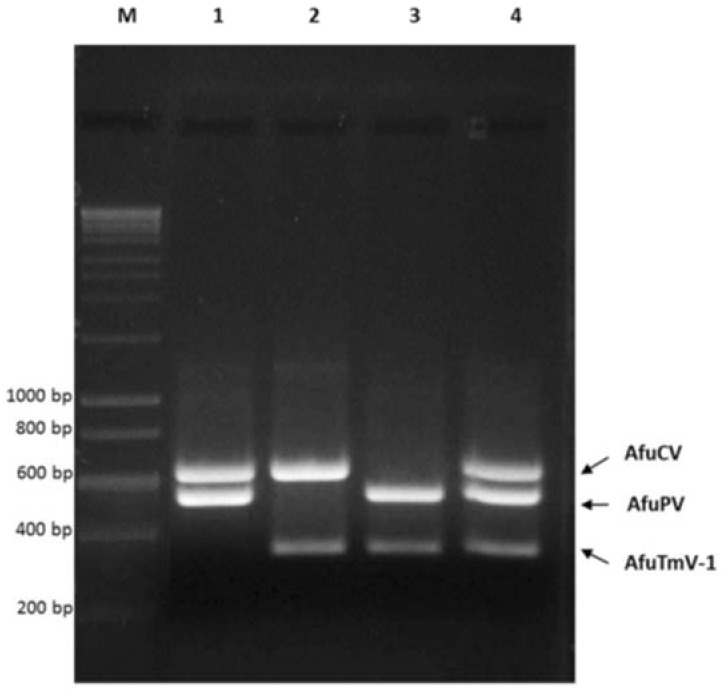
Simultaneous detection of *A. fumigatus* mycoviruses using multiplex RT-PCR. Mixed dsRNA combinations were used to perform multiplex RT-PCR. In lanes 1, 2 and 3, combinations of AfuCV and AfuPV-1, AfuCV and AfuTmV-1 and AfuPV-1 and AfuTmV-1, were used as template, respectively. In lane 4, dsRNAs belonging to three *A. fumigatus* mycoviruses were mixed and multiplex RT-PCR was performed using all three primer pairs. Hyperladder-I was used as a marker to estimate the size of the amplicon (Lane M).

Mixed infections using dual or triple combinations of dsRNA viruses were also amplified simultaneously using multiplex RT-PCR (Figure 3). Combinations of AfuCV and AfuPV-1, AfuCV and AfuTmV-1, AfuPV-1 and AfuTmV-1 and AfuCV, AfuPV and AfuTmV-1 were used as template and multiplex RT-PCR was performed using all three primer pairs. In all cases, amplicons were only generated from their respective mycovirus template RNAs. 

**Figure 4:**
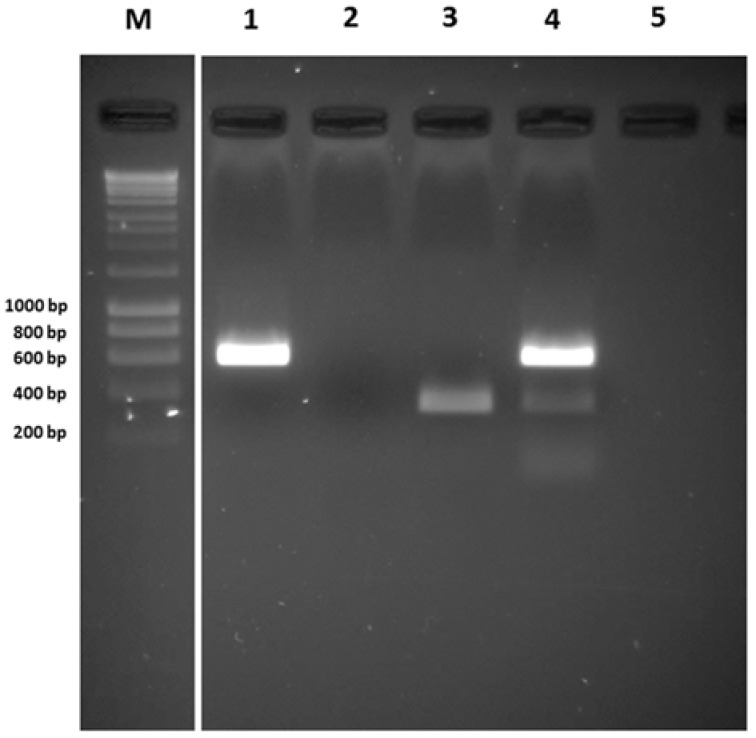
Simultaneous detection of mycoviruses in an *A. fumigatus* isolate (A80) which was infected naturally by a mixture of AfuCV and AfuTmV-1. In lanes 1, 2 and 3, amplicons were produced using AfuCV, AfuPV-1 or AfuTmV-1 primers, respectively. In lane 4, RNA isolated from the mixedly infected A80 isolate was used as template and multiplex RT-PCR was performed using all three primer pairs. Lane 5 showed the negative template control. Hyperladder-I was used as a marker to estimate the size of the amplicon (Lane M).

The efficiency of the multiplex PCR amplification assay was also tested using a naturally mixed infected *A. fumigatus* isolate A80 [5], which was naturally infected with a combination of AfuCV and AfuTmV-1 (Figure 4). Extracted RNA, as described above, was used as template for single PCRs including AfuCV, AfuPV-1 and AfuTmV-1 primers separately first. It was found that A80 gave amplicons of the correct sizes when AfuCV and AfuTmV-1 primer sets were used. However, as anticipated the A80 isolate did not give any amplicon when the AfuPV-1 primer set was used. When the multiplex PCR was conducted with all three primer sets the A80 isolate only gave amplicons with primers specific for AfuCV and AfuTmV-1. However, the amplicon generated from AfuTmV-1 was small in quantity as compared to that generated from AfuCV suggesting the former was present in lesser amounts in the mixedly infected A80 isolate (Figure 4, lane 4).

In addition, all positive amplicons from multiplex PCR were sequenced in order to confirm the specificity of PCR products. The sequences were blasted to the deposited GenBank sequences to confirm the specificity of the PCR amplified DNA fragments. Sequences of amplified products were identical to the *RdRP* gene sequences of respectively AfuCV, AfuPV-1 and AfuTmV-1.

## 4. Discussion

It is essential to develop a rapid and reliable method to detect *A. fumigatus* mycoviruses. This study developed a multiplex PCR assay for rapid detection of three *A. fumigatus* mycoviruses (AfuCV, AfuPV-1 and AfuTmV-1) using specific primers for each mycovirus.

There are various studies on *Aspergillus* mycoviruses, especially ones infecting the medically important human pathogen *A. fumigatus*. In these studies, variable mycovirus incidence was reported in different *A. fumigatus* populations using common nucleic acid extraction methods [4,5,6]. Recently, a simple and rapid method has been developed to purify viral dsRNA from plant and fungal tissues using cellulose powder [22]. There is no study reported on rapid detection of *Aspergillus* mycoviruses except those using small-scale dsRNA extraction approaches [23]. Even though viruses can be detected with the small-scale dsRNA extraction method, it can be laborious where large numbers of samples are to be screened and when the virus is in low titre and sensitivity is a problem. Conversely, multiplex RT-PCR amplification provides sensitivity and specificity in diagnosis of virus infection along with the other advantages such as being easy to perform, rapid and cost-efficient [10]. Recently, a number of novel techniques to detect mycoviruses, record their incidence and characterize them have been developed. These include RT-LAMP [12] and next generation sequencing-mediated virus detection and characterization [24,25], and it is conceivable that such techniques could be combined with multiplex-PCR technology in virus detection and characterization in the future.

Additionally, the method of RNA extraction is of great importance as is one of the factors determining the efficiency of multiplex PCR. The method developed in this study uses the RNA template obtained using a rapid kit extraction method which also makes the procedure quicker and reproducible. 

To our knowledge, this is the first study reporting multiplex RT-PCR optimized for mycoviruses. However, it is only applicable to characterized viruses that have a known genomic sequence. It is also possible that novel mycoviruses in *A. fumigatus* can be missed as this method has only been developed based on sequence information of known *A. fumigatus* mycoviruses. In order to overcome this problem, a primer pair could be designed based on the conserved sequence (if there is any) of known *A. fumigatus* mycoviruses assuming that the undiscovered *A. fumigatus* mycoviruses also have this conserved sequence. This primer pair could be added to the multiplex PCR in order not to miss any potential novel mycoviruses. Since it is known that all viruses, especially those with RNA as their genome, evolve and mutate, it is entirely feasible that the multiplex PCR amplification protocol as described might have limited application in the future. However, we have been careful in our design of oligonucleotides used in the protocol such that they are in conserved regions of the genomes, mutations to which are likely lethal for virus replication. Multiple detection by deep-sequencing is also a very useful approach to screen and furthermore elucidate the viromes [25,26]. In conclusion, a novel multiplex RT-PCR assay which is rapid, reliable and cost-effective was developed to detect AfuCV, AfuPV-1 and AfuTmV-1 infection of *A. fumigatus* isolates. The multiplex RT-PCR assay developed here would be useful for the studies on determining the incidence of known *A. fumigatus* mycoviruses and to establish a system for a comprehensive survey of them. Moreover, it could be useful to detect mycovirus infection rapidly in order to design treatment to fungal infection as mycoviruses can alter fungal pathogenicity.

## Figures and Tables

**Table 1:** Oligonucleotide primers used for multiplex polymerase chain reaction (PCR) to detect virus infection in *A. fumigatus*. AfuCV: Aspergillus fumigatus chrysovirus; AfuPV-1: Aspergillus fumigatus partitivirus-1; AfuTmV-1: Aspergillus fumigatus tetramycovirus-1.

Virus	Primer ID	Sequence 5′→3′	Tm (°C)	Amplicon Size and Positions in Genomes	GenBank No
AfuCV	MCV1F MCV1R	TCGACACAGAAGGCGATATG CGCCGTTGATAAAAGTCCAT	63.8 63.6	592 bp (1114–1705)	FN178512.1
AfuPV-1	MPV1F MPV1R	TCAGCTGGAGCCACCTTTAT CTCCACTTCTGAGCATCACG	63.7 63.7	497 bp (546–1042)	FN376847.3
AfuTmV-1	MTmV1F MTmV1R	AACCAGGACGTCGTTTCCTTCG AACAGTGTATTGAGGGTGTC	64.7 63.3	328 bp (953–1280)	HG975302
